# Self-Reported Consumption of Bottled Water v. Tap Water in Appalachian and Non-Appalachian Kentucky

**DOI:** 10.13023/jah.0502.04

**Published:** 2023-08-01

**Authors:** Jason W. Marion

**Affiliations:** Eastern Kentucky University, jason.marion@eku.edu

**Keywords:** Appalachia, Race, Bottled Water, Tap Water, Water Safety, Drinking Water, Kentucky, Water Infrastructure, Water Aesthetics

## Abstract

**Introduction:**

Quantitative studies on drinking water perceptions in Appalachia are limited. High-profile water infrastructure failures in the U.S. and Eastern Kentucky, coupled with human-made and natural disasters in the Appalachian Region, have likely impacted opinions regarding tap water.

**Purpose:**

To use existing unexplored data to describe baseline tap water v. bottled water consumption in Kentucky.

**Methods:**

Telephone-based cross-sectional data were obtained from the 2013 Kentucky Health Issues Poll (KHIP) directed by the Foundation for a Healthy Kentucky. Among many items in KHIP, self-reported consumption of bottled water over tap water, reasons for bottled water use, and demographic data were obtained.

**Results:**

Among Appalachian (n=356) and non-Appalachian (n=1,125) Kentucky respondents, a significantly higher frequency of Appalachian Kentuckians reported drinking bottled water more often than tap water relative to non-Appalachian Kentuckians (57% v. 34%; *X*^2^ p < 0.001). Appalachian residency significantly predicted bottled water consumption in simple and multivariable logistic regression adjusted for significant covariates (i.e., age, sex, and race). Among persons consuming bottled water more than tap water, Appalachian Kentuckians reported significantly more concerns regarding tap water taste or smell (*p* = 0.005) and safety (*p* = 0.008) than non-Appalachians.

**Implications:**

These results from 2013 data pre-date headline news items related to public water and likely underestimate current bottled water preferences. New data are needed, and these results warrant further investigation into tap water aesthetics in Appalachia, bottled water consumption impacts on personal finances, and approaches to build public trust for public drinking water among multiple populations including Appalachian Kentuckians.

## INTRODUCTION

The health-related quality of life is reduced in Central Appalachia with the all-cause mortality having risen 20.3% and 33.8% from 1999 to 2017 in the Central Appalachian states of Kentucky and West Virginia, respectively.^1^ In Kentucky and other states with Appalachian regions, these declines have been greatest in Appalachian counties and are linked to increases in “deaths of despair.”^1^ Cancer occurrence also remains higher in this region, particularly due to a number of smoking- and socioeconomic-related factors.^2^ Data from the 2021 Behavior Risk Factor Surveillance System indicate Kentucky and West Virginia have the highest percentages of persons self-rating their health as poor (7.2% and 7.0%, respectively).^3^ Nationally, persons with poor or fair self-rated health are 36% more likely to be concerned about health risks from environmental pollutants than persons with excellent or very good health;^4^ it is useful to characterize perceptions of environmental health factors in Central Appalachia since restoring and improving infrastructure has been proposed for improving health in the region.^1^

Across the U.S., 41% of Americans identify drinking water quality among their top three environmental concerns affecting their health.^4^ Accordingly, there are persistent concerns regarding drinking water quality and safety across Central Appalachia,^5^ including within Appalachian Kentucky.^6^ In Central Appalachia, public awareness exists regarding wastewater infrastructure challenges (straight-pipe pollution),^7^ environmental catastrophes (chemical spills,^8^ slurry spills,^5^,^9^,^10^ and mineral resources extraction.^5^,^9^–^11^ Furthermore, while studies evaluating environmental perceptions of drinking water in Central Appalachia exist, most of these studies have had small sample sizes or did not have proximal non-Appalachian reference groups.^6^

In an era when Central Appalachia’s perceptions of public and private drinking water systems have been impacted by awareness of straight-pipe pollution, environmental spills, mineral resources extraction,^5^–^11^ and at least three extreme flood events (all declared major federal disasters [DR-4663-KY, DR-4595-KY, DR-4358-KY]),^12^ conversations regarding infrastructure modernization and climate resiliency abound due to increased risks.^13^ For assisting infrastructure planning and influencing public trust in municipal systems, an understanding of existing public perceptions of drinking water in Appalachia are of value. One approach for ascertaining public opinion of local municipal and private drinking water is to examine reported bottled water consumption v. tap water use.^9^,^11^ In this study, an analysis was performed on self-reported bottled water consumption using data from the 2013 Kentucky Health Issues Poll (KHIP), directed by the Foundation for Healthy Kentucky and Interact for Health.

## METHODS

Data collected by the 2013 version of KHIP were downloaded from OASIS, a web-based tool administered by Interact for Health to provide access to public health and social science data. The 2013 data were used since the 2013 questionnaire contained questions regarding water consumption. While these data are 10 years old, no other known surveys on bottled water consumption or drinking water perceptions exist for comparing Appalachian and non-Appalachian Kentucky. The statewide questionnaire^14^ was administered by the Institute for Policy Research at the University of Cincinnati by telephone for understanding the opinions and practices of Kentuckians regarding multiple issues (smoking bans, Medicaid expansion, school nutrition, the Affordable Care Act, etc.), including their consumption of fruits, vegetables, and water. The approximately 20-minute confidential questionnaire provided respondents a $10 gift card for their time. The protocol for data collection and public use sharing of non-identifiable data was approved by the Institutional Review Board at the University of Cincinnati (#2013-6867).

The telephone-based survey aimed to obtain complete surveys from at least 300 randomly selected households from each of Kentucky’s five regions (Eastern Kentucky, Lexington, Louisville, Northern Kentucky, and Western Kentucky). The survey obtained 1,551 responses from 951 and 600 landline and cell phone responses, respectively, between October and November 2013. Approximately 22,500 phone numbers were called, yielding an overall response rate of 6.9%.^15^,^16^

Two questions, Q22 and Q23, inquired about drinking water sources. Q22 asked, “When you drink water, what type of water do you drink most often? Please think about the water that you drink in a typical day, whether this is at home, at work or anywhere else. Would you say you most often drink… tap water, bottled water, water from a well, or water from somewhere else?” Q22 responses were obtained categorically as either tap water, bottled water, water from a well, water from somewhere else, don’t know, or non-applicable/refused.

The questionnaire directed respondents who reported not drinking tap water to Q23, “What is the most important reason you do not drink tap water more often?” Q23 responses were recorded by the interviewer using pre-coded reasons listed as follows: “tap water isn’t’ safe”, “don’t like tap water appearance (dirty/cloudy/color/particles)”, “bugs/worms/bacteria in tap water”, “chemical or pesticide contamination (other than chlorine)”, “tastes or smells (include chlorine)”, and “makes me ill/sick to my stomach”. Other survey options—don’t know and non-applicable/refused—were available.

Demographic information was also provided by respondents. The instrument obtained county residence data, whereby respondents were coded in the available dataset as Appalachian or not in accordance with Appalachian Regional Commission designations. Birth county was obtained and coded as Appalachian or not Appalachian. Other demographic data and smoking history were also used from the KHIP dataset.

Data were analyzed using Stata 15. For comparing respondents who reported consuming bottled water more than tap water, binary coding was used. Respondents who reported drinking bottled water more than tap water were coded as “1” for bottled water user. Persons reporting to consume tap water more often were coded as “0”. Persons reporting to most often drink well water, water from somewhere else, were unsure, or preferred not to answer were not included in statistical tests.

In comparing bottled water users v. tap water users, cross tabulations with chi-square and Fisher’s exact tests were used for evaluating differences between groups. Exact tests were used when any cell count was below five observations. Associations with self-reported bottled water consumption were reported using crude odds ratios (cOR) and adjusted odds ratios (aOR) obtained through simple and multivariable logistic regression, respectively. The most parsimonious logistic regression model associated with bottled water consumption was generated through stepwise backward elimination of nonsignificant (*p* ≥ 0.05) covariates from a model inclusive of all covariates. Multivariable models were evaluated for model fit, discrimination, and potential multicollinearity using the Hosmer-Lemeshow goodness-of-fit test, the area under the receiver–operator curve (AUC), and uncentered variance inflation factor (VIF) values, respectively.

## RESULTS

A total of 1,474 (95%) of the 1,551 KHIP respondents reported drinking tap water or bottled water. Upon limiting the results to these tap water consumers (n = 893) and bottled water consumers (n = 581), 39.4% of respondents most often consumed bottled water. The 77 (5%) persons not reporting to most often consume bottled or tap water reported drinking well water (n = 35), water from somewhere else (n = 19), were unsure (n = 10), or preferred not to answer (n = 13).

Among 1,474 respondents most often consuming tap or bottled water, the majority were female (59%), had graduated or attended college (59%), were over 46 years of age (68%), had a smoking history (51%), were white (90%), had no children in the home (71%), had a financial status placing them at over 200% of the federal poverty level (59%), resided in non-Appalachian Kentucky (76%), and were born outside Appalachia (64%) (Table 1).

Among all of the covariates assessed, bottled water was reported to be consumed more often than tap water (>50%) among 18- to 29-year-olds, non-White respondents, households with one child, and among respondents residing in an Appalachian county.

Among all covariates, use of bottled water more often than tap water was highest among persons from Appalachian counties: 57% of respondents consume bottled water most often (Table 1). By comparison, non-Appalachian respondents had 34% of respondents consuming bottled water most often.

Several statistically significant cORs were observed related to certain demographic groups reporting to consume bottled water more often than tap water (Table 1). Specifically, there was an increased likelihood of bottled water consumption among females relative to males, persons without a high school degree relative to college graduates, current smokers relative to never smokers, non-White respondents relative to White respondents, respondents with two or more children in the household relative to respondents with no household children, and among those residing or born within Appalachian counties relative to non-Appalachian counites (Table 1).

Upon adjusting for all covariates in Table 1, multiple covariates that had significant cORs were no longer significantly associated with consuming bottled water more than tap water in the multivariable model. However, several covariates remained significant (*p* < 0.05). Using the aORs in Table 1, the odds of bottled water consumption more often than tap water was 78% greater among females than males, 107% greater among non-Whites than Whites, substantially greater among younger populations than those 65 years or older, and 130% greater among persons residing in Appalachian Kentucky v. non-Appalachian Kentuckians.

Overall, the saturated model in Table 1 provided acceptable discrimination (AUC = 0.70) and model fit whereby there was no difference between the observed and model-predicted results (*p* = 0.346). There were indications of a modest amount of multicollinearity with VIFs indicating a moderate correlation among predictors (mean VIF = 2.28, range: 1.13 – 4.10).

The most parsimonious multivariable model (Table 2) was based upon 1,438 responses. Upon removing non-significant terms, the Appalachian birth county met criteria for inclusion (*p* < 0.05) into the final model (Table 2). The final model demonstrates that the odds of consuming bottled water more often than tap water was 134% greater for persons residing in Appalachian counties than for those residing in non-Appalachian Kentucky; 32% greater for persons born in Appalachian Kentucky v. non-Appalachian Kentucky; 90% greater for non-White persons than White persons; 64% greater for females than males; and substantially more frequently for young adults and adults v. persons aged 65 years or over. In regard to age differences, persons in the 18- to 29-year-old age group had 257% greater odds of bottled water consumption than persons over 65 years of age. Similarly, persons aged 30 to 45 years and 46 to 64 years had 100% and 71% greater odds of bottled water consumption over tap water, relative to those aged 65 years or over.

An evaluation of model discrimination, fit, and potential for multicollinearity, demonstrated that the parsimonious model (Table 2) had less than acceptable discrimination (AUC = 0.672), excellent fit whereby there was no difference between the model predicted and observed results (p = 0.753), and no indications of issues with multicollinearity among covariates (mean VIF = 1.51, range: 1.10 – 1.88). While the overall model in Table 2 is significant (likelihood ratio Chisquare *p* < 0.0001), additional covariates and/or a larger dataset would be needed to enhance model discrimination (sensitivity and specificity) for predicting which respondents are consumers of bottled water more than tap water and vice versa.

The KHIP questionnaire enabled exploration of the top reason any respondent reported for why they consumed bottled water more often than tap water. Through this question, bad taste or smell was the top reason among respondents, representing 41% and 52% of bottled water consumers from non-Appalachian and Appalachian counties, respectively (Table 3). Overall, significant differences between respondents from Appalachian counties v. non-Appalachian counties were observed (Table 3). Specifically, Appalachian respondents were more likely to report drinking bottled water more than tap water because their tap water reportedly tasted or smelled bad (*p* = 0.005) or because they perceived their tap water as unsafe (*p* = 0.008). Kentuckians residing outside Appalachian counties were significantly more likely than Appalachian Kentuckians to indicate that having easy access to bottled water was their primary reason for consuming bottled water more than tap water (*p* = 0.010).

Table 2 demonstrates that non-White respondents in Kentucky were 90% more likely to consume bottled water than White respondents (aOR = 1.90; 95% C.I.: 1.31–2.74). In evaluating possible reasons for consuming bottled water more often than tap water, Table 4 demonstrates that non-White respondents consuming bottled water were significantly more likely to report doing so because of the perception that the water was not clean (*p* < 0.001). There were no other significant differences between non-White and White respondents regarding their primary reason for consuming bottled water more often than tap water.

When focusing on non-White participants in Appalachian counties, a total of 18 (13%) of the 142 non-White Kentuckians resided in Appalachian counties. Among these 18 respondents, nine (53%) of 17 consumed bottled water more than tap water, and one respondent did not provide water consumption information. Among non-White Appalachian respondents, their prevalence (53%) of bottled water consumption more often than tap water was not different from the prevalence among White Appalachians (191 [57%] of 335) consuming bottled water more often than tap water (*p* = 0.861).

## DISCUSSION

In 2013, 57% of persons residing within Appalachia consumed bottled water before tap water, which was significantly higher than non-Appalachian Kentuckians (34%). The prevalence observed in non-Appalachian Kentucky counties in 2013 corresponds with national data, in which approximately 33% of the U.S. population consumes bottled water over tap water.^17^ Since 2013, major regional and national events have likely adversely impacted public confidence in municipal/tap water. The National Health and Nutritional Examination Survey (NHANES) demonstrated statistically significant declines in tap water consumption among low socioeconomic and non-White populations from 2007 to 2016.^18^ Public opinion polling indicated that 35% of the U.S. in 2018 believed drinking water quality was worsening,^17^ and public concern regarding municipal water may be greater now than in 2013 due to awareness of national news stories spanning the Flint Water Crisis, to public system failures in Jackson, Mississippi and at least one community in Eastern Kentucky.^19^

Water safety and taste/smell were identified among Appalachian Kentuckians as a reason for bottled water consumption over tap water. While private sources supplying home taps may be of shared or greater concern among residents, Appalachian Kentucky has more drinking water violations per community water system than most of the U.S.^20^ Regions with more drinking water violations among their community water systems have 14% more bottled water sales per capita corroborating the KHIP results. In Appalachia, additional concerns arise as some community members prefer to drink untreated environmental waters, including roadside springs, because of perceptions of better taste, trust, quality, and/or health reasons. These waters are perceived as safer despite evidence of fecal contamination.^5^,^21^

Several small studies have corroborated public confidence concerns in their water.^5^,^9^,^10^ In a small study (n = 23) in a coal-impacted Appalachian region, most respondents (82.6%) reported a lack of trust in their home tap water.^5^ The inability to trust tap water has resulted in most homes in the region purchasing distilled and bottled water, with 57% of respondents in this study using bottled water over tap water. This prevalence of bottled water use in socioeconomically disadvantaged areas presents financial concerns including greater water costs as well as time and travel costs linked to purchasing and collection^10^,^11^.

Public health issues are also raised when tap water concerns are so pronounced. Research has suggested that promoting increased water consumption and decreased consumption of sugar-sweetened beverages is difficult in Appalachia,^22^ and some evidence suggests it is more difficult when water safety is a concern.^23^ There is also added public health relevance for persons searching out and collecting water from springs and wells, as microbiological and chemical contaminants can be present in unregulated, unmonitored, environmental water^5^,^21^,^24^ and improperly used storage containers.^25^,^26^

Research indicates restoring public trust in drinking water systems can be difficult. Following a chemical spill into the Elk River in Charleston, West Virginia, 80% of persons would not drink tap water months after the spill-related advisory was lifted.^8^ Ideas to restore trust from Eastern Kentucky’s Shaping Our Appalachian Region Health Work Group recommend increased testing of private wells and springs, enhanced source water protections and evaluations, and expansion or improvement in public water infrastructure.^6^ In the northern West Virginia community of Morgantown, perceptions regarding taste and safety as well as water consumption increased with point-of-use water filters; however, filter adoption tended to be higher among households with higher socioeconomic status, indicating a need for further study on methods to improve filter adoption, such as reduced filter costs and understanding filter costs v. bottled water costs.^11^

### Limitations

The considerable amount of non-response to the random telephone survey limits generalizability of the results. For example, among respondents, 62% were women, 71% had no children in the home, and 18% and 32% of respondents from Appalachian and non-Appalachian counties had college degrees, respectively. Given these demographics, the respondents in this study are significantly different than the general population in Kentucky. However, if a non-response bias existed for gender or household children, it may have been non-differential between Appalachian and non-Appalachian counties, in which there were no significant differences in their proportion of female respondents (*p* = 0.952) and those without children in the home (*p* = 0.147). As with crosssectional telephone surveys, there is potential for misclassification due to social desirability in responses regarding education, poverty status, and possibly even bottled water opinions.

## IMPLICATIONS

The evidence that bottled water consumption is more prevalent than tap water consumption in Appalachian Kentucky and in non-White communities is considerable. While these data are limited in that they are from 2013 and reliant on self-reports rather than direct observations, the trend from other studies suggests these results may underreport bottled water consumption in the region. The results indicate bottled water consumption in Appalachian parts of Kentucky likely far exceed levels in much of the U.S. Public policy needs are apparent. Public policies could be informed by exploring approaches and interventions which provide greater access and education pertaining to (1) point-of-use filters; (2) subsidizing bottled water purchases in areas lacking suitable public water infrastructure; and (3) providing greater monitoring resources for unregulated community and private drinking water sources. More current studies on tap water perceptions in Appalachia may be beneficial.

**SUMMARY BOX**

**What is already known about this topic?**

There is evidence from small studies that bottled water consumption is more prevalent among Appalachian households than tap water consumption, and that citizens in this region have concerns regarding the aesthetics and safety of tap water. The impacts of bottled water consumption on household financial resources and as an indicator of environmental concerns has been documented in this region and in minority communities around the U.S.

**What is added by this report?**

Studies on bottled water consumption v. tap water consumption in Appalachia are limited to small studies (< 50 households) and/or studies lacking a reference population outside the region. Using data obtained by the Kentucky Health Issues Poll, comparisons related to bottled water v. tap water consumption were able to be made between Appalachian and non-Appalachian Kentuckians while also adjusting for potential confounding factors like age, sex, and educational status.

**What are the implications for future research?**

The results illustrate significant disparities in bottled water consumption and potential inequities regarding the provision or acquisition of quality potable water between Appalachian Kentucky and other parts of Kentucky and the United States. The results and analysis add to, corroborate, and strengthen the existing body of literature justifying policy actions for enhancing water quality monitoring programs, water-related infrastructure, access, affordability and/or trust regarding potable water for household use in Appalachia.

## Figures and Tables

**Table 1:** Respondent demographics including the prevalence of consuming bottled water more often than tap water along with crude and adjusted odds ratios for predicting bottled water consumption

Covariate (No. Missing)	Study Pop. n (%)	Bottled Water n (%)	cOR (95% C.I.)*	aOR (95% C.I.)†
Sex of Respondent (77)				
Male	553 (36)	187 (34)	Referent	Referent
Female	921 (59)	394 (43)	***1.46 (1.18–1.82)*** §	***1.78 (1.37–2.31)***
Education Status (85)				
Less than H.S.	148 (10)	62 (42)	Referent	Referent
High School	458 (31)	207 (45)	1.14 (0.78–1.66)	1.38 (0.86–2.19)
Some College	427 (29)	173 (41)	0.94 (0.65–1.38)	0.87 (0.54–1.41)
College Graduate	433 (30)	136 (31)	**0.64 (0.43–0.93)**	0.74 (0.44–1.23)
Respondent Age (97)				
18 to 29 years	167 (11)	92 (55)	***3.19 (2.19*** **–** ***4.62)***	***4.21 (2.61–6.78)***
30 to 45 years	277 (19)	121 (44)	***2.01 (1.47*** **–** ***2.76)***	***2.18 (1.41–3.39)***
46 to 64 years	582 (39)	241 (41)	***1.84 (1.40*** **–** ***2.40)***	***1.79 (1.28–2.48)***
65 years or over	428 (29)	119 (28)	Referent	Referent
Smoking Status (83)				
Current smoker	322 (22)	152 (47)	**1.47 (1.13–1.92)**	1.20 (0.86–1.66)
Previous smoker	431 (29)	157 (36)	0.94 (0.74–1.21)	1.18 (0.87–1.59)
Never smoked	715 (49)	270 (38)	Referent	Referent
Respondent Race (99)				
White	1312 (90)	503 (38)	Referent	Referent
Non-White	140 (10)	70 (50)	**1.61 (1.13** ***–*** **2.28)**	***2.07 (1.38*** **–** ***3.10)***
Household Children (88)				
None	1035 (71)	374 (36)	Referent	Referent
One	198 (14)	103 (52)	1.92 (1.41***–***2.60)	1.37 (0.95***–***1.99)
Two or More	230 (16)	100 (43)	*1.36 (1.01 - 1.82)*	0.99 (0.67***–***1.47)
Poverty Status (303)				
138% and below FPL	311 (25)	136 (44)	Referent	Referent
>138% and below 200%	201 (16)	88 (44)	1.00 (0.71–1.43)	1.17 (0.79***–***1.74)
>200% FPL	736 (59)	275 (37)	0.77 (0.59–1.00)	1.28 (0.92–1.78)
Appalachian County (77)				
Non-App county	1122 (76)	381 (34)	Referent	Referent
Appalachian county	352 (24)	200 (57)	***2.56 (2.00*** **–** ***3.27)***	***2.30 (1.66*** **–** ***3.17)***
County of Birth (77)				
Non-App county	941 (64)	328 (35)	Referent	Referent
Appalachian county	533 (36)	253 (47)	***1.69 (1.36 - 2.09)***	1.32 (0.99–1.75)

NOTES:

* Crude odds ratio with 95% confidence interval

† Adjusted odds ratio with 95% confidence interval, adjusting for all covariates in table.

§ Bold with italics represents *p* < 0.001; bold alone represents *p* < 0.01; and italics alone represents *p* < 0.05.

**Table 2:** The most parsimonious multivariable logistic regression model with adjusted odds ratios for predicting the likelihood a Kentuckian consumes bottled water more often than tap water

Covariate	b	S.E._b_	aOR*	*p*
**Sex of Respondent**				
Male			Referent	
Female	0.492	0.12	1.64 (1.29–2.07)	<0.001
**Age of Respondent**				
18 to 29 years	1.271	0.2	3.57 (2.41–5.26)	<0.001
30 to 45 years	0.695	0.17	2.00 (1.44–2.79)	<0.001
46 to 64 years	0.538	0.14	1.71 (1.30–2.26)	0.001
65 years or over			Referent	
**Respondent Race**				
White			Referent	
Non-White	0.64	0.19	1.90 (1.31–2.74)	<0.001
**Appalachian County**				
Non-App county			Referent	
Appalachian county	0.849	0.15	2.34 (1.76–3.11)	<0.001
**County of Birth**				
Appalachian county	0.276	0.13	1.32 (1.02–1.70)	0.036
**Constant**	−1.63	0.15		

NOTES:

* Adjusted odds ratio with 95% confidence interval

**Table 3:** The primary reason provided by respondents from non-Appalachian and Appalachian counties for why they consume bottled water more often than tap water

Top Reason for Bottled Water over Tap Water	Non-Appalachian County Respondent n/N (%)	Appalachian County Respondent n/N (%)	*p* *
Bad taste or smell	183/451 (41)	118/227 (52)	***0.005***
Easy access to bottled	87/451 (19)	26/227 (11)	***0.010***
Not safe	39/451 (9)	35/227 (15)	***0.008***
Chemicals in the water	46/451 (10)	21/227 (9)	0.696
Don’t have tap water	27/451 (6)	10/227 (4)	0.392
Not clean	19/451 (4)	6/227 (3)	0.306
Drink both bottled and tap	17/451 (4)	3/227 (1)	0.093
Not cold enough	11/451 (2)	1/227 (0.4)	0.070
Other	22/451 (5)	7/227 (3)	0.276
Don't know/refused	3/454 (0.7)	1/228 (0.4)	1.000

NOTES:

* χ^2^ or Fisher’s exact test *p*; Fisher’s exact test was used if any 2 x 2 table cell count was less than 5.

**Table 4:** The primary reason provided by White and non-White respondents for why they consume bottled water more often than tap water

Top Reason for Bottled Water over Tap Water	White Respondent n/N (%)	Non-White Respondent n/N (%)	*p* *
Bad taste or smell	233/503 (46)	29/70 (41)	0.441
Easy access to bottled	98/503 (19)	10/70 (14)	0.298
Not safe	56/503 (11)	9/70 (13)	0.670
Chemicals in the water	50/503 (10)	9/70 (13)	0.452
Don’t have tap water	15/503 (3)	0/70 (0)	0.236
Not clean	9/503 (2)	8/70 (12)	***<0.001***
Drink both bottled and tap	14/503 (3)	3/70 (4)	0.450
Not cold enough	8/503 (2)	0/70 (0)	0.605
Other	18/503 (5)	1/70 (3)	0.494
Don't know/refused	2/503 (0.5)	1/70 (1)	0.324

NOTES:

* χ^2^ or Fisher’s Exact Test *p*; Fisher’s Exact Test was used if any 2 × 2 table cell count was less than 5.
